# Distribution, clinicopathologic features and survival of breast cancer subtypes in Southern China

**DOI:** 10.1111/j.1349-7006.2012.02339.x

**Published:** 2012-07-04

**Authors:** Cong Xue, Xi Wang, Roujun Peng, Yanxia Shi, Tao Qin, Donggen Liu, Xiaoyu Teng, Shusen Wang, Li Zhang, Zhongyu Yuan

**Affiliations:** 1 State Key Laboratory of Oncologyin in South ChinaGuangzhou, China; 2 Department of Medical Oncology, Sun Yat-Sen University Cancer CenterGuangzhou, China; 3 Department of Breast Oncology, Sun Yat-Sen University Cancer CenterGuangzhou, China

## Abstract

Breast cancer research and treatment by different subtypes is an inevitable trend. We investigated the clinicopathologic features and outcomes of different breast cancer subtypes in Southern China. A total of 5809 patients with invasive ductal carcinomas were identified. Immunohistochemical (IHC) markers for estrogen receptor (ER), progesterone receptor (PR), Her2/neu, and Ki-67 proliferation index were used to classify cases into five molecular subtypes. Clinicopathologic characteristics and survival rates were analyzed retrospectively. Of all patients, 31.1% were luminal A subtype, 30.4% luminal B (high Ki-67), 13.1% luminal B (Her2/neu+), 9.0% Her2/neu and 16.5% triple negative subtype. Luminal B (high Ki-67) presented primarily in premenopausal patients with the lowest average age (43.0 years). Her2/neu positive tumors were more closely associated with aggressive features including increased tumor size, positive lymph node status and lymphvascular invasion (LVI). Triple negative subtype was characterized by poorer histologic grade. Her2/neu positive cases had presented the worst 5-year disease-free survival (DFS) and overall survival (OS). Multivariate analyses of OS and DFS suggested that there were different negative prognostic factors for the five subtypes. The benefit of the cyclophosphamide, methotrexate, and 5-fluorouracil (5FU) (CMF) regimen was equal to that of anthracycline-based and Taxane-based regimens for patients with luminal A subtype and triple negative subtype, but inferior to anthracycline-based and Taxane-based regimens for those with two luminal B subtypes and Her2/neu subtype. The prognostic significance of traditional markers may differ among subtypes. This study revealed the distinct clinicopathologic characteristics, systemic therapy benefits, prognostic factors and survival rate among different breast cancer subtypes.

Breast cancer is the most common malignancy for women in China, as well as in other countries.^1^ Although the incidence of breast cancer is lower in China than that in other countries, it has increased by 80% in young women in the past two decades.^2^ In addition, because of the far-reaching base of Chinese inhabitants, the new cases of breast cancer accounted for 21.3% of all newly diagnosed cases of breast cancer in the world.^3^

Breast cancer is known to be a heterogeneous disease, which exhibits distinct clinical presentations, aggressiveness, response to treatments, and outcomes among different types of patients with breast cancer or ethnic populations.^4^ Gene expression profiling analysis has categorized breast cancer into specific subtypes with different clinical outcomes, including luminal A, luminal B, Her2 overexpressed and basal-like subtypes.^4^,^5^ Luminal A subtypes were shown to be associated with elderly postmenopausal patients and to have a better prognosis than patients with luminal B. Her2 overexpressed and basal-like subtypes appeared to be more aggressive and had inferior survival advantage.^6^

Immunohistochemical (IHC) has been validated as a surrogate for molecular gene profiling in several studies.^4^,^7^,^8^ Furthermore, in 2009, Cheang *et al*.^9^ separated luminal A with Ki-67 index >14% as luminal B. Those patients with hormone receptor (HR) positive status and higher Ki-67 expression (luminal B [high Ki-67]) were believed to have relatively poor outcomes similar to those with both HR and Her2 positive phenotype (luminal B [Her2/neu+]). Using the IHC methodology, which can easily gauge the characteristics of tumors and predict patient appreciable responses to treatments and overall outcomes, this method is commonly used in daily clinical practice. Several population-based studies investigated the clinico-pathologic characteristics in different molecular subtypes, and the results showed marked variance among various ethnic populations.^4^,^10^–^14^ Thus, the prevalence of intrinsic subtypes must be taken into account when analyzing survival data, and when screening patients for global clinical studies.^14^

However, until now, there was little information regarding the distribution of breast cancer subtypes among Chinese women with breast cancer. Moreover, luminal B subtypes actually comprise high Ki-67 proliferation index and Her2 positive phenotypes; none articulated any clinical differences among them. Therefore, we investigated the distribution, clinicopathologic features and survival rates of patients with various breast cancer subtypes as classified by IHC. In addition, the independent prognosis factors for each of these subtypes were further identified in this study.

## Materials and Methods

### Study population

**Fig. 1:**
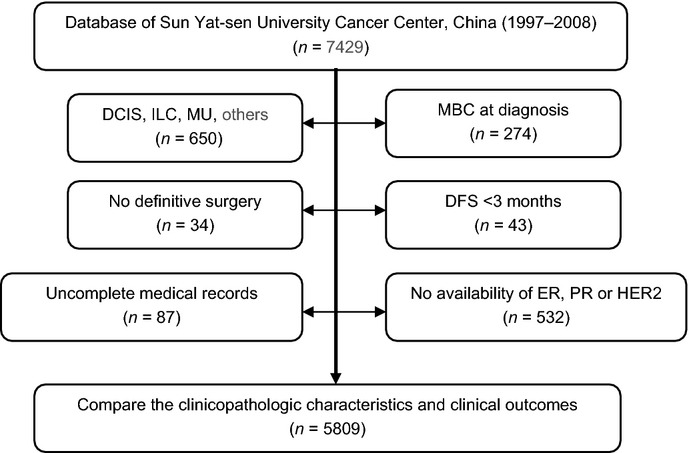
Patient inclusion in the study. DCIS, ductal carcinoma *in situ*; DFS, disease-free survival; ER, estrogen receptor; ILC, invasive lobular carcinoma; MBC, metastatic breast cancer; MU, mucoid adenocarcinoma; PR, progesterone receptor; other rare types including phyllodes tumor, adenoid cystic carcinoma, neuroendocrine carcinoma, metaplastic carcinoma and lymphoma of breast cancer.

This population-based study included a cohort of women with newly diagnosed breast cancer treated in a single, comprehensive cancer center, Sun Yat-Sen University Cancer Center, between January 1997 and December 2008. A total of 7429 participants were involved in the study. The exclusion criteria included: (i) metastatic disease at diagnosis (*n =* 274); (ii) without definitive surgery (*n =* 34); (iii) incomplete medical records and follow-up status (*n =* 87); (iv) disease-free survival (DFS) less than 3 months (*n =* 43); (v) unavailable for estrogen receptor (ER), progesterone receptor (PR) or Her2 status, retrospectively (*n =* 532); (vi) pathologic type for ductal carcinoma *in situ* (DCIS) (*n =* 216), invasive lobular carcinoma (ILC) (*n =* 111) and mucinous adenocarcinoma (MA) (*n =* 122), other rare types including phyllodes tumor (*n =* 42), adenoid cystic carcinoma (31), neuroendocrine carcinoma (35), metaplastic carcinoma (56) and lymphoma (37) (Fig. 1). All pathologic results in this study were histopathologically warranted by two separate experienced pathologists according to diagnostic criteria. The pathologic information was obtained from the pathologic department of Sun Yat-Sen University Cancer Center and explicitly documented. In total, 5809 patients were eligible with invasive ductal carcinoma (IDC) in this study.

All patients were staged according to the American Joint Committee on Cancer (AJCC 2010, 7th edition) TNM Staging System for Breast Cancer. The study was reviewed and approved by the Institutional Review Board and academic committee of Sun Yat-Sen University Cancer Center.

### Initial treatments

Among 5809 electable participants, 5541 (95.4%) received radical mastectomy and 268 (4.6%) underwent breast-conserving surgery. Of 5130 (88.3%) received adjuvant chemotherapy following surgery. The regimens mainly consisted of three types: classical cyclophosphamide, methotrexate, and 5-fluorouracil (5FU) (CMF) regimen, anthracycline-based regimen, and combined anthracycline and taxane regimen (termed as taxane-based regimen). The median cycle of chemotherapy is 5 (range, 2–10). The main indications for radiotherapy include the following: the number of positive lymph nodes >4 in the lymphatic region, selective for patients with one to three positive lymph nodes; the size of primary tumor >5 cm; breast-conserving surgery. In our study, indications for three chemotherapy were confirmed on the basis of NCCN guideline development. Chemotherapy was recommended for all patients recruited in the study in the exception for ER/PR-positive women with stage I breast cancer. CMF regimen was recommended to all of eligible patients recruited before 2002, while anthracycline-based regimen including cyclophosphamide, epirubicin, and 5-fluorouracil (5FU)/cyclophosphamide, adriamycin, and 5-fluorouracil (CEF/CAF) was optimal for participants after 2002 owing to real superiority relative to CMF based on several randomized-control trials. According to the NCCN guideline, taxane-based regimen was preferred for those participants with high risk factors, including ER/PR negative status and positive lymph nodes. Totally, all chemotherapy regimens were set up by an experienced medical oncologist.

### Immunohistochemistry

Tumors from all patients were assessed or reassessed (if the initial results were already available) for ER, PR, Her2 status by two experienced pathologists, and Ki-67 proliferation index in a central reference laboratory in Sun Yat-sen University Cancer Center. With respect to HR determined by IHC, the percentage of neoplastic cells expressing either ER or PR was recorded. Fewer than 5% of neoplastic cells were considered as negative for HR expression.^15^ Her2 was determined initially by IHC and graded from 0 to 3+. Her2 negative was defined as Her2 graded 0 or 1+, positive defined as 3+. Scores of Her2 2+ were confirmed either as Her2 negative or Her2 positive according to the FISH analysis.^16^

In this study, we classified breast cancer into five subtypes based on the expression of ER, PR, Her2 and Ki-67 proliferation index. Luminal A: ER and/or PR+, Her2−, and low Ki-67 proliferation index (≦14%); luminal B (high Ki-67): ER and/or PR+, Her2−, and high Ki-67 index (>14%); luminal B (Her2/neu+): ER and/or PR+, Her2+, and any Ki-67; Her2/neu subtype: ER and PR−, Her2/neu+, and any Ki-67; triple negative subtype (TN): ER, PR and Her2 all negative, and any Ki-67.^17^

### Statistical analysis

Biostatistical analysis was accomplished by a statistical group comprised of three experienced statisticians. Additionally, professional statistical software was also applied in our study to make a guarantee for the reliability and convincibility. DFS was defined as the interval from the first treatment for breast cancer to the first recurrence (locoregional relapse, distant metastasis, or contralateral breast). OS was calculated as the period from the date of diagnosis to the date of death from any cause or the date of the last follow-up. Locoregional relapse was defined as the recurrence of either the treated breast or the ispilateral lymph node bearing area (axillary, internal mammary, supraclavicular node).

Clinico-pathologic parameters were assessed among five subgroups by the χ^2^ test. Cumulative survival probabilities were calculated through the Kaplan–Meier method. Survival rates were compared by log-rank test. Multivariate analyses were performed by the Cox regression model. Some traditional prognostic factors, such as tumor size, lymph node involvement, stage, HR status, Her-2 status, lymphvascular invasion (LVI), and Ki-67 proliferation index were included in the multivariate analysis with enter model. Hazard ratio (HR) was presented with their 95% confidence intervals (CI). All statistical tests were two-tailed and *P* < 0.05 was considered significant. Statistical analysis was performed by spss 16.0 (SPSS Inc., Chicago, IL, USA).

## Results

### Distribution and clinicopathologic features of breast cancer subtypes

Of 5809 eligible patients, 1805 (31.1%) were classified as luminal A subtype, 1765 (30.4%) as luminal B (high Ki-67), 760 (13.1%) as luminal B (Her2/neu+), 522 (9.0%) as Her2/neu and 957 (16.5%) as TN subtype. The clinicopathologic characteristics of patients with breast cancer recruited into this study are shown in Table 1.

**Table 1:** Clinical characteristics and treatment of different breast cancer subtypes

Variables	All cases	Luminal A	Luminal B (high KI-67)	Luminal B (Her2/neu+)	Her2/neu	TN	*P-*value
No. (%)	5809	1805 (31.1%)	1765 (30.4%)	760 (13.1%)	522 (9.0%)	957 (16.5%)	
Age							
Mean ± SD (years)	47.5 ± 10.7	51.0 ± 11.2	43.0 ± 9.7	47.5 ± 9.6	47.9 ± 9.9	47.4 ± 10.6	<0.0001
Age-specific groups, *n* (%)							
35	752 (12.9)	152 (8.4)	321 (18.2)	80 (10.5)	72 (13.8)	127 (13.3)	<0.0001
36–69	4859 (83.6)	1534 (85.0)	1412 (80.0)	671 (88.3)	455 (85.2)	797 (83.3)	
70	198 (3.4)	119 (6.6)	32 (1.8)	9 (1.2)	5 (1.0)	33 (3.4)	
Menopausal status *n* (%)							
Premenopausal	3347 (57.6)	723 (40.1)	1289 (73.0)	389 (51.2)	313 (60.0)	633 (66.1)	<0.0001
Postmenopausal	2462 (42.4)	1082 (59.9)	476 (27.0)	371 (48.8)	209 (40.0)	324 (33.9)	
Tumor size, *n* (%)							
2.0 cm	1744 (30.0)	608 (33.7)	555 (31.4)	205 (27.0)	121 (23.2)	255 (26.6)	<0.0001
2.0–5.0 cm	3630 (62.5)	1090 (60.4)	1109 (62.8)	475 (62.5)	331 (63.4)	625 (65.3)	
>5.0 cm	435 (7.5)	107 (5.9)	101 (5.7)	80 (10.5)	70 (13.4)	77 (8.0)	
Lymph node status, *n* (%)							
0	2876 (49.5)	1039 (57.6)	849 (48.1)	265 (34.9)	210 (40.2)	513 (53.6)	<0.0001
1–3	1568 (27.0)	475 (26.3)	459 (26.0)	251 (33.0)	139 (26.6)	244 (25.5)	
4–9	799 (13.8)	176 (9.8)	273 (15.5)	135 (17.8)	90 (17.2)	125 (13.1)	
>10	566 (9.7)	115 (6.4)	184 (10.4)	109 (14.3)	83 (15.9)	75 (7.8)	
AJCC stage group, *n* (%)							
Stage I	1080 (18.6)	771 (21.6)	342 (19.4)	86 (11.3)	59 (11.3)	164 (17.1)	<0.0001
Stage II	3248 (55.9)	1989 (55.7)	937 (53.1)	408 (53.7)	277 (53.1)	574 (60.0)	
Stage III	1481 (25.5)	810 (22.7)	486 (27.5)	266 (35.0)	186 (35.6)	219 (22.9)	
Hormonal receptor status							
ER+	3601 (62.0)	1587 (87.9)	1496 (84.8)	518 (68.2)	0 (0.0)	0 (0.0)	<0.0001
PR+	3897 (67.1)	1644 (91.1)	1602 (90.8)	651 (85.7)	0 (0.0)	0 (0.0)	
ER+/PR+	4330 (74.5)	1805 (100.0)	1765 (100.0)	760 (100.0)	0 (0.0)	0 (0.0)	
HER2 status							
Positive	1282 (22.1)	0 (0.0)	0 (0.0)	760 (100.0)	522 (0.0)	0 (0.0)	<0.0001
Negative	4527 (77.9)	3570 (100.0)	1765 (100.0)	0 (0.0)	0 (0.0)	0 (0.0)	
Histologic grade, *n* (%)							
I	1455 (24.9)	792 (43.9)	482 (27.3)	59 (7.8)	38 (7.3)	74 (7.7)	<0.0001
II	1869 (32.2)	690 (38.2)	624 (35.4)	293 (38.6)	138 (26.4)	124 (13.0)	
III	2495 (43.0)	323 (17.9)	659 (37.5)	408 (53.7)	346 (66.3)	759 (79.3)	
Ki-67, *n* (%)							
15%	1901 (32.7)	1625 (90.0)	0 (0.0)	91 (12.0)	45 (8.6)	140 (14.6)	
>15%	3610 (62.1)	0 (0.0)	1765 (100.0)	629 (82.8)	443 (84.9)	773 (80.8)	
Unknown	298 (5.1)	180 (5.0)	0 (0.0)	40 (5.3)	34 (6.5)	44 (4.6)	<0.0001
LVI, *n* (%)							
Yes	157 (2.7)	38 (2.1)	44 (2.5)	27 (3.6)	25 (4.8)	23 (2.4)	0.164
No	5652 (97.3)	1767 (97.9)	1721 (97.5)	733 (96.4)	497 (95.2)	934 (97.6)	
Primary surgery							
Mastectomy	5541 (95.4)	1728 (95.7)	1677 (95.0)	726 (95.5)	498 (95.4)	912 (95.3)	0.001
BCS	268 (4.6)	77 (4.3)	88 (5.0)	34 (4.5)	24 (4.6)	45 (4.7)	
Adjuvant chemotherapy							
Yes	5130 (88.3)	1459 (80.8)	1588 (90.0)	722 (95.0)	490 (93.9)	871 (91.0)	0.001
No	679 (11.7)	346 (19.2)	177 (10.0)	38 (5.0)	32 (6.1)	86 (9.0)	
Adjuvant chemotherapy type							
CMF regimens	670 (11.5)	273 (15.1)	181 (10.3)	58 (7.8)	29 (5.6)	128 (13.4)	<0.0001
Anthracycline-based	2896 (49.9)	789 (43.7)	932 (52.8)	373 (49.1)	289 (55.4)	513 (53.6)	
Taxen-based	1564 (26.9)	397 (22.0)	475 (26.9)	290 (38.2)	172 (33.0)	230 (24.0)	
No	679 (11.7)	346 (19.2)	177 (10.0)	38 (5.0)	32 (6.1)	86 (9.0)	
Adjuvant radiotherapy							
Yes	1354 (23.3)	343 (19.0)	457 (25.9)	211 (27.8)	136 (26.1)	207 (21.6)	<0.0001
No	4455 (76.7)	1642 (81.0)	1308 (74.1)	549 (72.2)	386 (73.9)	750 (78.4)	
Adjuvant endocrine therapy							
ER antagonist	3671 (63.2)	1474 (81.7)	1479 (83.8)	632 (83.2)	29 (5.6)	102 (10.7)	<0.0001
Aromatase inhibitor	374 (6.4)	130 (7.2)	122 (6.9)	57 (7.5)	4 (0.8)	16 (1.7)	
No	1764 (30.4)	201 (11.1)	164 (9.3)	71 (9.3)	489 (93.7)	839 (87.7)	

AJCC, American Joint Committee on Cancer; BCS, breast-conserving surgery; CMF, cyclophosphamide, methotrexate, and 5-fluorouracil (5FU); ER, estrogen receptor; PR, progesterone receptor; SD, standard deviation; TN, triple negative subtype.

There were significant differences among breast cancer subtypes according to the mean age at diagnosis. The mean age was lowest in the luminal B (high Ki-67) group (43.0 years) and highest in the luminal A group (51.0 years). When stratified by age bands, five subtypes also differed significantly (*P* < 0.0001): the highest percentage of patients at the age of ≤35 were in luminal B (high Ki-67) subtype (18.2%); luminal B (Her2/neu) had the highest percentage in the age group 35–69 (88.3%), while luminal A subtype had the highest proportion of patients with an age of ≥70 (6.6%). Interestingly, only 3.4% of patients with TN subtype were aged ≥70.

Over half of the cases were premenopausal patients (57.6%). Luminal B (high Ki-67) had the highest percentage of premenopausal patients (73.0%), followed by TN subtype (66.1%). 59.9% of patients with luminal A subtype were postmenopausal, taking the upper hand from five subtypes of breast cancer.

A significant difference was found when breast cancer subtypes were compared in terms of tumor size (*P* < 0.0001). The highest proportion of tumors with diameter >5 cm was detected in Her2/neu and luminal B (Her2/neu) subtypes (13.4% and 10.5% respectively), while tumors ≤ 2 cm were observed with greater frequency in luminal A subtype (33.7%).

We observed the highest percentage of negative lymph node cases in luminal A (57.6%) and TN (53.6%) tumors; in contrast, patients with the Her2/neu positive status, including luminal B (Her2/neu+) and Her2/neu subtypes, had the highest prevalence of positive lymph node status (65.1% and 59.8%, respectively).

Regarding the AJCC stage, significant differences were also observed, with the highest percentage of stage I/II tumors in luminal A (77.3%) and TN (77.1%) subtypes, whereas luminal B (Her2/neu+) and patients with Her2/neu tumors showed the largest prevalence of stage III cancers (35.0% and 35.6%, respectively).

When participants were categorized by the histologic grading system, we observed a significant difference among breast cancer subtypes (*P* < 0.0001): grade III tumors were more frequent in TN and Her2/neu positive tumors, including luminal B (Her2/neu+) and Her2/neu subtypes (79.3%, 53.7% and 66.3%, respectively).

The biomolecular subtypes differed significantly with regards to the LVI (*P =* 0.004). Her2/neu subtype showed the highest prevalence of LVI (4.8%), whereas luminal A tumors presented the lowest level of LVI prevalence (2.1%).

When HR status was stratified according to age, there was no difference between patients aged <50 years and those aged ≥50 years (75.0% *vs* 73.8%). Although the percentage of PR positive was slightly higher in patients aged <50 than those aged ≥50 (69.1% *vs* 63.5%, *P* < 0.001), a significant difference was not observed.

All patients underwent local and/or systemic treatments. The therapeutic strategy was determined by a multidisciplinary team, including oncosurgeon, medical oncologist and radiologist. Local management included surgery and radiotherapy. Systemic treatments included chemotherapy and endocrine therapy. About 95% of patients received mastectomy. Surgical approaches, breast-conserving surgery and mastectomy, were not significantly different among the cohorts of different breast cancer subtypes. However, significant difference was noted in adjuvant radiotherapy and systemic therapy. The proportion of adjuvant chemotherapy and radiotherapy was obviously lower in luminal A than that in other subtypes. As expected theoretically, the HR positive cases received a higher proportion of adjuvant endocrine therapy. These results demonstrated that the choice of therapy was dependent on factors such as hormone receptor status, tumor size, lymph node status, and others involved in our study.

### Survival and breast cancer subtypes

**Fig. 2:**
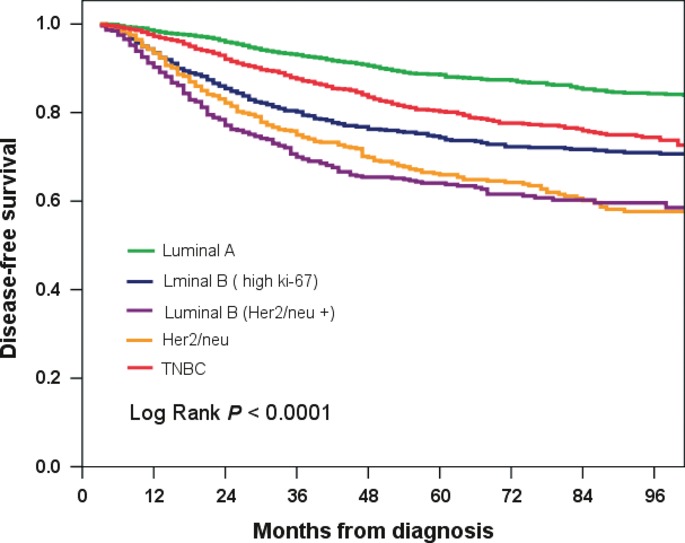
Kaplan–Meier disease-free survival by breast cancer subtypes. 5-year disease-free survival (DFS) rates of luminal A, luminal B (high Ki-67), luminal B (Her2/neu+), Her2/neu and triple negative breast cancer (TNBC) were 88.6%, 80.2%, 66.0%, 64.1%, and 74.1%, respectively (*P* < 0.0001).

**Fig. 3:**
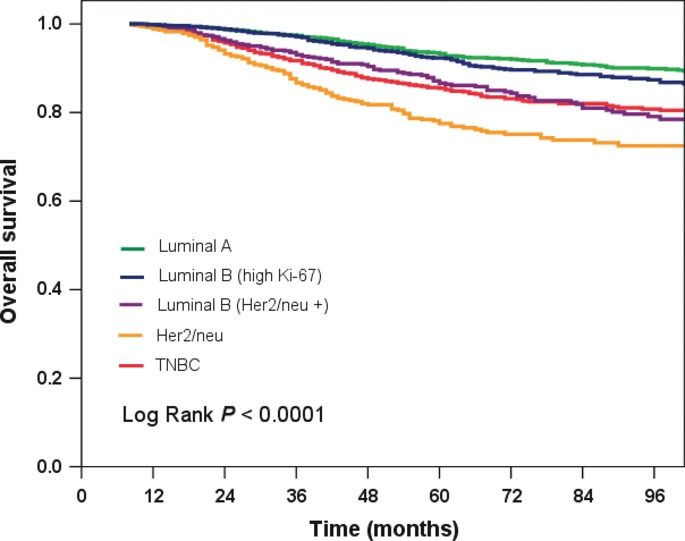
Kaplan–Meier overall survival by breast cancer subtypes. 5-year overall survival (OS) rates of luminal A, luminal B (high Ki-67), luminal B (Her2/neu+), Her2/neu and TN were 93.3%, 92.2%, 86.6%, 77.5%, and 85.5%, respectively (*P* = 0.0001).

The median time of follow-up was 60 months (14–162 months). With the follow-up, 988 (17.0%) patients presented relapsed, and 640 (11.0%) patients died from any tumor-related cause. The estimated 5-year DFS rate and OS rate of all patients were 78.6% and 89.3%, respectively. Five-year DFS rates of luminal A, luminal B (high Ki-67), luminal B (Her2/neu+), Her2/neu and TN were 88.6%, 80.2%, 66.0%, 64.1%, and 74.1%, respectively, (*P* < 0.0001, Fig. 2). The patients with Her2 positive tumors regardless of HR status exhibited a significant disadvantage for DFS. In parallel, 5-year OS rates were 93.3%, 92.2%, 86.6%, 77.5%, and 85.5% in luminal A, luminal B (high Ki-67), luminal B (Her2/neu+), Her2/neu and TN subtype, respectively, (*P* < 0.0001, Fig. 3).

### Effects of prognostic factors on DFS and OS in breast cancer subtypes

Various clinicopathologic variables were analyzed by univariate and multivariate analysis in the different subtypes of breast cancer. Totally, lower age, larger tumor size, lymph node positive status, AJCC stage III, negative HR status, Her2/neu positive status, histologic grade III, LVI, and high Ki-67 index were adverse prognostic factors for DFS and OS (Table 2).

**Table 2:** Multivariate analysis of disease-free survival and overall survival in all population

	Disease-free survival			Over survival		
		
Variables	HR	95% CI	*P-*value	HR	95% CI	*P-*value
Age, years (≤35 *vs* >35)	0.67	0.58–0.77	<0.0001	0.75	0.61–0.92	0.005
Tumor size, cm (≤ 2 *vs* >2)	1.82	1.55–2.14	<0.0001	1.83	1.46–2.31	<0.0001
Node status (Neg. versus Pos.)	1.46	1.25–1.70	<0.0001	1.45	1.16–1.83	0.001
Stage (I/II *vs* III)	1.81	1.56–2.09	<0.0001	2.21	1.80–2.70	<0.0001
Hormone receptor status (Neg. versus Pos.)	0.82	0.72–0.93	0.002	0.65	0.55–0.78	<0.0001
HER–2 status (Neg. versus Pos.)	1.15	1.11–1.18	<0.0001	1.09	1.05–1.14	<0.0001
Histologic grade (I/II *vs* III)	1.30	1.21–1.38	<0.0001	1.31	1.19–1.44	<0.0001
Vascular invasion (No versus Yes)	1.77	1.37–2.28	<0.0001	1.80	1.29–2.51	0.001
Ki–67 (14% *vs* >14%)	0.94	0.89–1.00	0.032	0.85	0.76–0.95	0.004

HR and 95% confidence intervals (CIs) were calculated using Cox regression analysis. HER-2, human epidermal growth factor receptor 2; HR, hazard ratio; Neg, negative; Pos, positive.

To evaluate the independent roles of adverse prognostic factors for DFS and OS for patients with different breast cancer subtypes, multivariate Cox regression analysis suggested that prognosis for different breast cancer subtypes was affected by different prognostic factors (Tables 3 and 4).

**Table 3:** Multivariate analysis of disease-free survival in different breast cancer subtypes

	Luminal A	Luminal B (high Ki-67)	Luminal B (Her2/neu+)	Her2/neu	Triple negative
					
Variables	HR (95% CI)	HR (95% CI)	HR (95% CI)	HR (95% CI)	HR (95% CI)
Age, years (≤35 *vs* >35)	0.75 (0.48–1.18)	0.55 (0.43–0.70)**	0.73 (0.50–1.07)	0.66 (0.45–0.96)*	0.80 (0.57–1.13)
Tumor size, cm (≤2 *vs* >2)	3.11 (2.00–4.84)**	1.99 (1.47–2.69)**	1.67 (1.18–2.36)**	1.00 (0.70–1.44)	2.16 (1.46–3.18)**
Node status (Neg. versus Pos.)	1.00 (0.69–1.45)	1.47 (1.09–1.98)*	1.19 (0.83–1.71)	1.57 (1.02–2.40)*	1.93 (1.38–2.71)**
Stage (I/II *vs* III)	1.68 (1.13–2.51)*	1.16 (0.87–1.54)	2.06 (1.50–2.82)**	1.79 (1.23–2.62)**	1.99 (1.45–2.73)**
Hormone receptor status (Neg. versus Pos.)	–	–	–	–	–
HER–2 status (Neg. versus Pos.)	–	–	–	–	–
Histologic grade (I/II *vs* III)	2.19 (1.84–2.59)**	1.21 (1.07–1.37)**	1.10 (0.97–1.26)	1.08 (0.92–1.27)	1.25 (1.03–1.52)*
Vascular invasion (No versus Yes)	2.65 (1.44–4.89)**	1.71 (1.02–2.84)*	1.21 (0.67–2.19)	2.21 (1.29–3.79)**	1.63 (0.86–3.08)
Ki–67 (14% *vs* >14%)	–	–	0.93 (0.82–1.06)	0.91 (0.71–1.17)	1.04 (0.82–1.31)

*P < 0.05; **P < 0.01. HR and 95% confidence intervals (CIs) were calculated using Cox regression analysis. HER-2, human epidermal growth factor receptor 2; HR, hazard ratio; Neg, negative; Pos, positive.

**Table 4:** Multivariate analysis of overall survival in different breast cancer subtypes

	Luminal A	Luminal B (high Ki-67)	Luminal B (Her2/neu+)	Her2/neu	Triple negative
					
Variables	HR (95% CI)	HR (95% CI)	HR (95% CI)	HR (95% CI)	HR (95% CI)
Age, years (≤35 *vs* >35)	0.53 (0.31–0.88)*	0.60 (0.42–0.85)**	1.06 (0.57–1.99)	0.73 (0.45–1.19)	0.95 (0.60–1.50)
Tumor size, cm (≤2 *vs* >2)	2.28 (1.33–3.90)**	1.82 (1.15–2.87)*	3.03 (1.52–6.06)**	1.22 (0.74–2.02)	1.75 (1.08–2.83)*
Node status (Neg. versus Pos.)	1.16 (0.70–1.94)	1.38 (0.91–2.11)	1.00 (0.54–1.83)	1.58 (0.88–2.83)	2.09 (1.32–3.32)**
Stage (I/II *vs* III)	1.99 (1.20–3.29)**	0.66 (0.33–1.31)	2.24 (1.36–3.69)**	2.21 (1.34–3.64)**	2.43 (1.61–3.66)**
Hormone receptor status (Neg. versus Pos.)	–	–	–	–	–
HER–2 status (Neg. versus Pos.)	–	–	–	–	–
Histologic grade (I/II *vs* III)	2.30 (1.84–2.88)**	1.19 (0.98–1.43)	1.24 (0.99–1.56)	1.16 (0.93–1.45)	1.24 (0.97–1.58)
Vascular invasion (No versus Yes)	1.98 (0.90–4.38)	1.84 (0.89–3.80)	2.19 (1.05–4.57)*	1.92 (0.96–3.86)	0.94 (0.38–2.30)
Ki–67 (14% *vs* >14%)	–	–	0.81 (0.57–1.15)	0.47 (0.29–0.77)**	0.62 (0.41–0.94)*

*P < 0.05; **P < 0.01. HR and 95% confidence intervals (CIs) were calculated using Cox regression analysis. HER-2, human epidermal growth factor receptor 2; HR, hazard ratio; Neg, negative; Pos, positive.

For luminal A subtype, large primary tumor, pathologic stage III and histologic grade III were independently unfavorable for benefits of DFS and OS, LVI only for DFS, and age at diagnosis only for OS.

For luminal B subtype (high Ki-67), younger age at diagnosis and large primary tumor were independently unfavorable factors for DFS and OS; and lymph node positive, histologic grade III and LVI only for DFS.

For luminal B subtype (Her2/neu+), large primary tumor and pathologic stage III were independently unfavorable factors for DFS and OS; and LVI only for OS.

For Her2/neu subtype, pathologic stage III was independently unfavorable factors for DFS and OS, younger at diagnosis, lymph node positive, and LVI only for DFS, high Ki-67 only for OS.

For TN subtype, large primary tumor, lymph node positive, and pathologic stage III were independently unfavorable factors for DFS and OS; histologic grade III only for DFS; and high Ki-67 only for OS.

### Effects of adjuvant chemotherapy on DFS in breast cancer subtypes

**Fig. 4:**
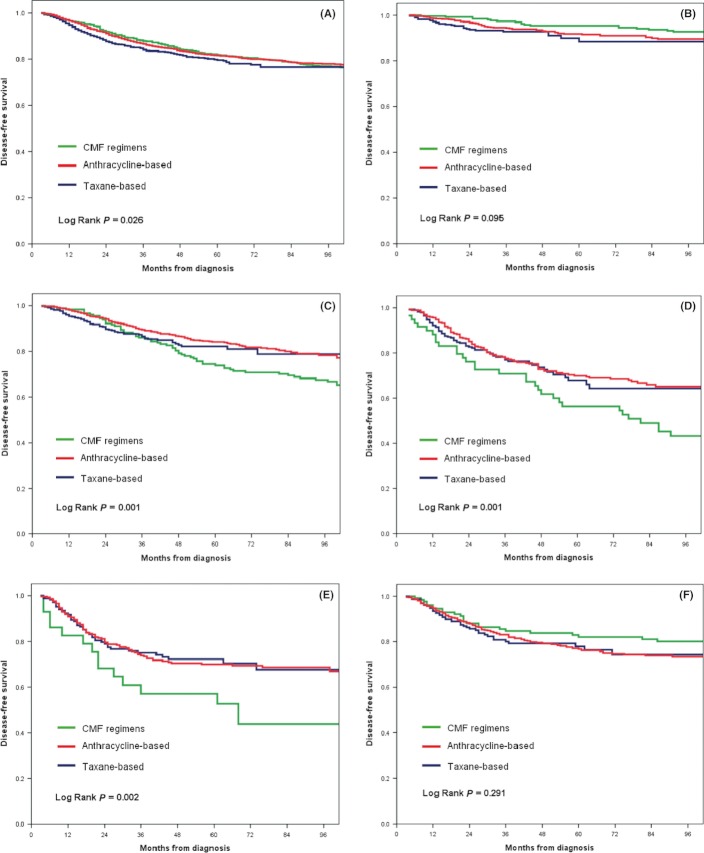
Kaplan–Meier disease-free survival by different chemotherapy regimens. (A) all population, anthracycline-based and Taxane-based regimens were superior to classical cyclophosphamide, methotrexate, and 5-fluorouracil (5FU) (CMF) regimens for disease-free survival (DFS) in all populations, there was no difference between anthracycline-based and Taxane-based regimens (*P* = 0.026); (B) luminal A, there was no difference among three regimens (*P* = 0.095); (C) luminal B (high Ki-67); (D) luminal B (Her2/neu+); (E) Her2/neu+, anthracycline-based and Taxane-based regimens were superior to classical CMF regimens for DFS in luminal B (whether high Ki-67 or Her2/neu, also Her2/neu, *P* = 0.001), but there was no difference between anthracycline-based and Taxane-based regimens (*P* = 0.126); (F) triple negative breast cancer (TNBC), there was no difference among all three regimens (*P* = 0.089).

For early breast cancer, the primary role of adjuvant chemotherapy can eliminate the occult micrometastasis to reduce the relapse. So we analyzed the relationship between adjuvant chemotherapy and recurrence in different breast cancer subtypes. In addition, most of the patients who did not perform adjuvant chemotherapy, presented some favorable factors, such as older at diagnosis, and hormone receptor positive, Her2/neu negative, small primary tumor, lymph node negative and so on. Subsequently, we further analyzed the effects of various adjuvant chemotherapy regimens in the breast cancer subgroups. Our results suggested three paradigms: first, anthracycline-based and Taxane-based regimens were superior to classical CMF regimens for DFS in all populations. Second, classical CMF regimen was not inferior to Non-CMF regimens for patients with luminal A subtype and triple negative subtype. Third, classical CMF regimen was significantly inferior to non-CMF regimens for patients with luminal B and Her2/neu subtypes. There was no significant different between anthracycline-based regimens and taxane-based regimens regardless of breast cancer subtypes (Fig. 4).

Patients with positive HR significantly benefited from endocrine therapy. However, in contrast, those with negative hormone receptor did not benefit from endocrine therapy (data not shown). The number of patients with the administration of aromatase inhibitors was in such a small number that we cannot analyze the effects between aromatase inhibitors and ER antagonists. Of 1282 patients with Her2/neu positive status, only 73 patients were treated with a high-titer monoclonal antibody against Her2/neu (trastuzumab), the 5-year DFS rate was significantly higher than those without trastuzumab treatment (72.8% *vs* 64.9%, *P* < 0.001), but still lower than those in the luminal A group (data not shown).

## Discussion

Recently, evidence has accumulated to support the notion that breast cancer subtypes are distributed in an alternative pattern corresponding to race or ethnicity.^4^,^15^,^18^ Among Asians, the proportion of Koreans with Her2/neu positive cancer was higher (36%) than that for Japanese (19%) and Chinese (26%),^19^ which was similar to that reported in this clinical study (22%). It was uncovered that the percentage of TN breast cancer varied from 10.2% to 30.6%,^4^,^5^,^14^,^15^,^20^,^21^ in which about 18.5% of patients were diagnosed with the TN subtype in China,^11^ consistent with our data. It indicated that the heterogeneity of genome profiles may have had a great contribution to the efficacy of breast cancer treatments. To maintain the consistency for analysis of our clinical data, our study comprised a larger number of IDC women with Han nationality. Additionally, a consecutive 10-year patient recruitment and a median 5-year follow-up were accomplished in the study.

With regard to breast cancer subtypes distribution in the recruited population, we reported the clinical data of two separate luminal B subtypes, HR positive status with high Ki-67 index and Her2/neu expression, respectively, for the first time. Significant differences were shown not only in the clinic-pathologic features, but also in DFS or OS rates and prognostic factors specific for breast cancer subtypes. Surprisingly, luminal B (high Ki-67 and Her2/neu+) took up to 43.5% in all, followed by luminal A (31.1%), TN (16.5%) and Her2/neu (9.0%). The luminal B (high Ki-67) were previously included into the luminal A subtype. However, with the new categorization, the results were not revealed as before. Previous studies had presented the patients with TN subtypes as the lowest mean age.^4^,^11^,^12^,^15^ In our study, we have demonstrated that patients with luminal B (high Ki-67) subtype actually had the lowest mean age. It was assumed the character of the luminal B (high Ki-67) was concealed by a larger proportion of luminal A with former categorization criteria.

The clinicopathologic characteristics and survival demonstrated that the new category was essential to improving outcomes in clinical practice. Considering only age distribution, the average age of breast cancer patient among Chinese was lower than that of northern America and Europe (mean 47.5 years),^4^,^12^–^14^ which was similar to Korea and Japan.^21^ Luminal B (high Ki-67) tumors arose in even younger patients than those with TN tumors (mean 43.0 years *vs* 47.4 years). The latter had similar age bands compared with those with Her2/neu positive subtypes. A certain proportion of TN patients were at the age of 70 years More than half of the patients were premenopausal, which correlated to the lower age mentioned above.

As for its aggressive property, Her2/neu overexpressed subtypes had a higher percentage of tumors with a large diameter (>5.0 cm), a larger percentage of lymph node involvement and LVI. Accordingly, patients with these subtypes were diagnosed more frequently with AJCC stage III than patients with other subtypes (*P* < 0.0001).

Although TN subtype had a relatively smaller tumor size, fewer lymph nodes involvement and less LVI, it had the poorest differentiation (*P* < 0.0001). Although controversy existed in view of the lymph node conditions of patients with TN tumors, most studies were consistent with our results,^13^,^15^,^21^,^22^ while one reported that the TN subtype had a more common lymph node involvement.^11^ Our study showed, as previously reported, that the TN subtype was more likely to have a higher Ki-67 proliferation index than others (*P* < 0.0001).^12^,^13^,^22^ Surprisingly, although the percentage of PR positive tumors was higher in the older patients,^23^,^24^ the percentage of positive HR status did not obviously increase with age at diagnosis. Moreover, the proportion of positive HR status was lower than that reported by Clark *et al*.^25^ It was unclear whether this is due to ethnic differences or other factors.

Our results demonstrated that an immunohistochemical panel of four biomarkers (ER, PR, HER2, and Ki-67) had a significant value in predicting the clinical outcomes of patients with breast cancer. Luminal A cohorts had the most favorable benefit for DFS and OS rates, followed by luminal B (high Ki-67), and TN. Her2/neu positive patients, including luminal B (Her2/neu) and Her2/neu subtypes, had the lowest survival rates, which is consistent with previous studies.^17^,^26^,^27^,^28^ In view of these results, our conclusions were listed as follows: first, HR positive cases have a generally better clinical outcome, which may imply HR positive is the predominant factor affecting survival.^26^ Second, it is important to distinguish the luminal B subtype from the luminal A subtype using Ki-67. There is a significant difference in clinical outcome between luminal B (high Ki-67) and luminal A subtypes. Finally, Her2/neu positive tumors present an aggressive tendency, and pose a high risk for the relapse of breast cancer. The prognosis for patients with TN subtype proceeds to a median level of DFS and OS.

As a whole, our results suggested that some traditional prognostic factors, such as age at diagnosis, primary tumor size, lymph node status, AJCC stage III, HR status, Her2/neu status, histologic grade III, and LVI were ascribed to adverse prognostic factors for DFS and OS, regardless of breast cancer subtypes. However, considering the different subtypes of breast cancer, there were distinct prognostic factors specific for each subtype. Age at diagnosis and histologic grade mainly affected the prognosis of populations with HR positive subtypes including luminal A and B (high Ki-67). Primary tumor size played an important prognostic role for all breast cancer subtypes except for Her2/neu subtype. The possible explanation was that patients with a Her2-positive smaller tumor size still had a potential risk of relapse.^27^,^28^ Interestingly, nodal status mainly affected the prognosis of patients with HR negative subtypes including Her2 positive and TN subtypes. Also, AJCC staging served as an important prognostic role, excluding luminal B (high Ki-67) subtype. LVI was an adverse prognostic factor primarily for DFS of the cases involving hormone receptor subtypes. Of note, Ki-67 index was a more complicated prognostic factor, which was associated with hormone receptor status. Our results showed that the high level of Ki-67 expression in breast cancers significantly increased the risk of relapse for HR positive breast cancer subtype (luminal A and luminal B), and risk of death for HR negative subtypes (Her2/neu and TN). Recent meta-analysis reported a statistically significant association between high Ki-67 expression and increased risk of breast cancer relapse and tumor-related death.^29^,^19^ It indicated that traditional bimolecular markers may not be an accurate prognostic factors for individual subtype, and further investigation is deemed necessary to be warranted.

Adjuvant chemotherapy has been a component of adjuvant therapeutic strategy for women with breast cancer owing to the observation that adjuvant chemotherapy yielded a significant trend toward the benefit for DFS and OS.^30^ As compared with single-agent chemotherapy, significant progression-free survival (PFS) and OS benefits resulted from CMF polychemotherapy.^31^ The incorporation of anthracycline agents and taxane including docetaxel and paclitaxel into adjuvant paradigm has been permitted for further improvements in spite of potential risk for increasing adverse effects.^30^,^32^ However, the diverse effects of breast cancer subtypes were infrequently referred and discussed. Furthermore, evidence was growing for the notion that breast cancer subtypes affect the biologic properties of malignancy.^17^ Notably, we observed that the effects of adjuvant chemotherapy on patients with breast cancer subtypes were significantly different. Our results showed that classical CMF regimen was not inferior to anthracycline/taxane-based chemotherapy regimens for patients with luminal A and TN subtypes. In consensus, it was previously reported that CMF regimen contributed to similar benefits for DSF and OS to anthracycline/taxane-involved regimen in patients with TN subtypes.^33^,^34^ However, due to no chance for endocrine therapy, 12 chemocycles of AC-T regimen was well-established for decreasing the risk of breast cancer recurrence,^17^ implying that long-term persistent infusion of taxane/anthracycline-involved regimen was optimal for subpopulations with TN breast cancer. In contrast, for patients with high Ki-67 or Her2/neu positive breast cancer, CMF regimen was remarkably inferior to anthracycline/taxane-based regimens, but there was no significant difference in the therapeutic efficacy between anthracycline-based and taxane-based regimens regardless of breast cancer subtypes. For patients with Her2/neu-positive breast cancer, trastuzumab (herceptin) was recommended as first line treatment for a significant improvement in DFS and OS of patients with early or late stage breast cancers.^17^,^19^ In our study, a high rate of trastuzumab-refusal may underestimate the efficacy of adjuvant chemotherapy. Why did the addition of taxane agents into adjuvant chemotherapy paradigm not further improve the prognosis of breast cancers? A possible explanation was that most of the regimens in our study comprised taxanes and anthracyclines, called the AT regimen. As reported previously, it was uncovered that the AT regimen did not improve DFS or OS compared with AC regimen in patients with operable breast cancer, and sequential administration of taxane after anthracycline-based therapy was more effective than that of concurrent administration.^17^,^34^ Despite ongoing debate, our clinical data partially reflected the discrepancy of breast cancer subtypes using IHC may affect the favorable efficacy of adjuvant chemotherapy.

However, we recognized some limitations of this clinical trial. First, this study was a retrospective analysis that lessened the credits as compared with a prospective randomized trial. Second, it was possible that the intergroup heterogeneity of the cohort of patients with five breast cancer subgroups accounted for a statistical bias of clinical data analysis. Third, further rationalization for therapeutic protocol was deemed necessary, in particular for trastuzumab application in patients with Her2/neu positive breast cancer. Despite these limitations, we still believed that the study was representative of the demographic characteristics of breast cancer subtypes in China, because all patients were selected from the Sun Yat-sen University Cancer Center, which was a well-respected comprehensive cancer center; over 60% of breast cancer patients in Southern China were treated in this hospital.

In conclusion, our results from a population-based data exhibited that breast cancer subtypes classified by IHC biomarkers presented the detectable differences in clinicopathologic characteristics, adjuvant radio-chemotherapy efficacy, prognostic factors and survival (DFS and OS) for patients with breast cancer subtypes in China. Rather than the TN subtype, luminal B (high Ki-67) was the lowest age at diagnosis, luminal B (Her2/neu) and Her2/neu subtypes had relatively poorer prognosis and an increased risk of relapse. A randomized treatment-control trial is warranted to further investigate the effects of IHC biomolecular markers on clinical outcomes in clinical practice. Meanwhile, our conclusions should be taken into account when analyzing the results of global clinical trials, and when conducting studies in the future.
